# Research on Corn Leaf and Stalk Recognition and Ranging Technology Based on LiDAR and Camera Fusion

**DOI:** 10.3390/s24165422

**Published:** 2024-08-22

**Authors:** Xueting Hu, Xiao Zhang, Xi Chen, Lu Zheng

**Affiliations:** Mechanical Engineering Training Centre, College of Engineering, China Agricultural University, Beijing 100083, China; huxueting@cau.edu.cn (X.H.); x.zhang@cau.edu.cn (X.Z.); chenxi2219@cau.edu.cn (X.C.)

**Keywords:** corn leaves and stalks, multi-sensor fusion, recognition and distance measurement

## Abstract

Corn, as one of the three major grain crops in China, plays a crucial role in ensuring national food security through its yield and quality. With the advancement of agricultural intelligence, agricultural robot technology has gained significant attention. High-precision navigation is the basis for realizing various operations of agricultural robots in corn fields and is closely related to the quality of operations. Corn leaf and stalk recognition and ranging are the prerequisites for achieving high-precision navigation and have attracted much attention. This paper proposes a corn leaf and stalk recognition and ranging algorithm based on multi-sensor fusion. First, YOLOv8 is used to identify corn leaves and stalks. Considering the large differences in leaf morphology and the large changes in field illumination that lead to discontinuous identification, an equidistant expansion polygon algorithm is proposed to post-process the leaves, thereby increasing the average recognition completeness of the leaves to 86.4%. Secondly, after eliminating redundant point clouds, the IMU data are used to calculate the confidence of the LiDAR and depth camera ranging point clouds, and point cloud fusion is performed based on this to achieve high-precision ranging of corn leaves. The average ranging error is 2.9 cm, which is lower than the measurement error of a single sensor. Finally, the stalk point cloud is processed and clustered using the FILL-DBSCAN algorithm to identify and measure the distance of the same corn stalk. The algorithm combines recognition accuracy and ranging accuracy to meet the needs of robot navigation or phenotypic measurement in corn fields, ensuring the stable and efficient operation of the robot in the corn field.

## 1. Introduction

Agricultural robots, as a new type of agricultural equipment integrating mechanization, automation, digitalization, and intelligence, aim to assist or replace humans in performing highly repetitive and labor-intensive tasks. These robots have shown excellent performance in various fields, such as corn planting and weeding. Environmental perception, a core function of agricultural robots, mimics human principles and capabilities of perceiving the environment, thus enabling the robot to perform complex operational functions [1]. Object detection technology plays a crucial role in environmental perception, where accurate measurement of characteristic parameters of targets, such as corn stalks, leaves, and tassels, is an indispensable component.

Early research primarily focused on monocular cameras and other visual sensors for measurement, as these provide rich image information, are cost-effective, and have low maintenance costs. However, the inherent sensitivity of single visual sensors to environmental conditions and the inaccuracy of spatial information often fail to ensure measurement accuracy and real-time performance [2,3]. Consequently, current field object detection technology increasingly relies on multi-sensor fusion as the main development direction.

LiDAR (light detection and ranging), another commonly used environmental perception sensor, offers more possibilities for agricultural robots. Compared to cameras, LiDAR provides accurate three-dimensional measurement information and is largely unaffected by complex lighting conditions in agricultural environments, demonstrating excellent anti-interference capabilities [4,5]. However, LiDAR point cloud data are sparse, and its ability to classify targets is relatively poor. Although expensive high-line-count multi-line LiDAR can increase point cloud density, it is not conducive to the widespread adoption of agricultural robots [6,7].

The fusion of LiDAR and camera holds promising application prospects in the field of target recognition for agricultural robots. This combined sensor approach leverages the complementary strengths of both sensors, enabling the target recognition and even tracking systems to better adapt to the complex lighting conditions and crop planting situations in the field. This results in more precise identification of corn characteristics, laying a solid foundation for subsequent tasks such as path planning and phenotypic detection.

Currently, research on environmental perception and object detection using LiDAR and depth camera fusion technology is primarily focused on the field of automotive autonomous driving. These studies typically aim to enhance vehicle navigation in complex urban environments, improve obstacle avoidance capabilities, and enable real-time detection of pedestrians and other vehicles. However, the application of this technology in agricultural scenarios is relatively limited, and its use in cornfields is even more scarce, with related research and practice still in the early stages. This limitation also highlights the vast potential for future developments in agricultural automation within cornfields. Huang et al. proposed a novel fusion network called EPNet, which enhances point features point-by-point and employs a consistency enforcement loss mechanism to address the issue of differing confidence levels in localization and classification during multi-sensor data fusion [8]. Bai et al. tackled the problems of traditional fusion methods being susceptible to complex and variable lighting conditions and low sensor alignment accuracy by using soft association extrema [9]. They utilized convolutional neural networks to predict the bounding boxes of targets from point clouds and adaptively fused camera image information, thereby improving the robustness and effectiveness of the fusion strategy. As a pre-fusion approach, the aforementioned strategies demand high real-time performance, resulting in high computational power requirements for the robotic systems.

Huang et al. first used convolutional neural networks for object detection on images and then clustered the target point clouds within the generated conical region based on projection relationships, resulting in three-dimensional bounding boxes of targets [10]. They achieved 3D target frame matching using appearance features, thereby implementing post-fusion of LiDAR and the camera. Similarly, Xu et al. first used YOLOv4 to detect objects in images, projected the point clouds onto the images, selected the target point clouds based on the 2D detection boxes, and then processed the target point clouds using an improved four-neighborhood clustering algorithm to generate point cloud bounding boxes [11]. After matching and fusion processing, they obtained the final object detection results, which are of practical significance for object detection and path planning tasks in complex robotic scenarios. Post-fusion algorithms demand less computational power from robots and are less dependent on network models, making them more suitable for complex and variable environments. Many scholars have made local improvements to image detection algorithms such as YOLO. Yue et al. [12] proposed a citrus recognition model YOLOv8-MEIN based on the improved YOLOv8n to address the problems of large overlap of citrus fruits, occlusion of branches and leaves, large number of parameters of existing models, and high computational complexity in complex environments. The comprehensive performance has been improved, providing a reference for the automated harvesting of citrus. To address the issues of low detection accuracy and poor robustness in apple detection, Du et al. [13] proposed an apple detection model based on an improved YOLOv8. This model enhances the accuracy and precision of apple detection, making it better suited to meet the demands of practical production. Local improvements have better recognition effects in specific situations, but agricultural scenes are highly unstructured and the objects to be detected vary greatly, so local improvements cannot adapt to complex and changing scenes.

**Figure 1:**
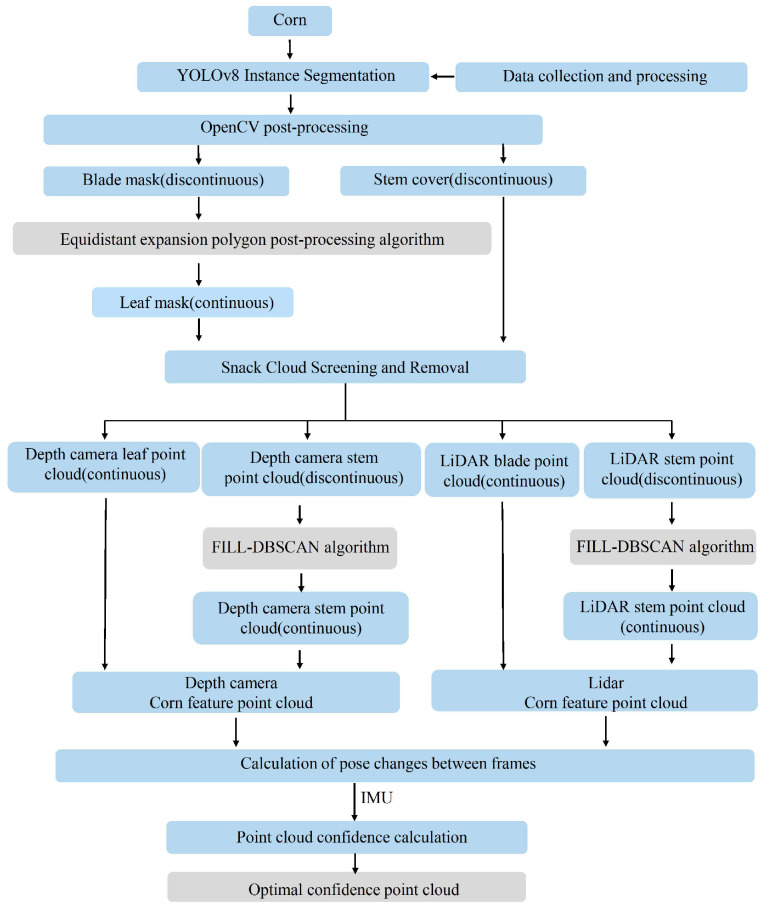
Flowchart of the algorithm.

In response to the current state of research, this paper first proposes a post-processing algorithm for corn leaf recognition based on equidistantly expanded polygons, enhancing the recognition accuracy of corn leaves with different morphologies. Secondly, it introduces a corn leaves and stalks distance measurement algorithm based on “optimal confidence point cloud” and achieves high-precision distance measurement of corn stalks with the assistance of the “FILL-DBSCAN” clustering algorithm. This enables accurate recognition and distance measurement of surrounding corn leaves and stalks during the operation of field robots in cornfields. After field tests, the equidistant expansion polygon post-processing algorithm can increase the maximum and average values of leaf recognition completeness to 97.1% and 86.4%. The average error of the optimal confidence point cloud for measuring the distance between corn leaves and stems is 2.9 cm, which has higher accuracy than that of single sensor measurement. The FILL-DBSCAN algorithm can also effectively realize the clustering of stems. Figure 1 illustrates the algorithm flowchart of this study.

## 2. Principle

In order to achieve high-precision recognition and ranging of corn leaf and stalk features, the coordinate systems of the depth camera and multi-line lidar are first converted to align the measurement data of the two sensors [14,15]. Secondly, the YOLOv8 model is trained through data acquisition and processing to realize the recognition and instance segmentation of corn leaves and stalks in the two-dimensional plane, and OpenCV post-processing is performed to reduce the influence of noise on the recognition accuracy. Due to the different curling degrees of leaves and the occlusion effect of leaves on the stalks, the YOLOv8 instance segmentation result is a discontinuous leaf and stalk mask. Then, the discontinuous leaf mask is post-processed using the equidistant expansion polygon post-processing algorithm, and the clustering of discontinuous leaves is achieved by using the overlapping effect between the appropriately expanded leaf contours to achieve complete leaf recognition. Then, the three-dimensional point cloud of the depth camera and lidar is screened by the complete leaf contour and incomplete stalk contour in the two-dimensional plane, and the redundant point cloud outside the contour is deleted to improve the calculation speed. At this time, the three-dimensional point cloud comes from two sensors, the leaf point cloud is continuous, while the stem point cloud is discontinuous. The FILL-DBSCAN algorithm is used to perform planar projection clustering on the discontinuous stem point cloud to achieve clustering of different sections of the same plant, thus realizing the screening of complete corn leaf and stem point clouds. Finally, the inter-frame pose transformation of the depth camera and LiDAR point clouds is calculated, and the point clouds are fused after introducing IMU data to determine the point cloud confidence of the two sensors to obtain the optimal confidence point cloud, thus completing the distance measurement of corn leaves and stems.

### 2.1. Sensor Data Fusion and FOV Matching

The prerequisite for the fusion of depth camera and LiDAR data is the temporal and spatial synchronization of the sensors. Using the lower-frequency sensor as the reference, temporal synchronization is achieved using timestamps. The internal parameters of the depth camera are calibrated, and after aligning the depth image with the color image, the external parameters of the two sensors are jointly calibrated to achieve spatial synchronization. This process also determin.es the relative pose between the depth camera and the LiDAR [16,17]. The calibration between the 3D point cloud and the 2D pixel coordinate system involves solving parameters. Assuming the real physical point in 3D space has coordinates (*X_L_*, *Y_L_*, *Z_L_*) in the LiDAR coordinate system and (*X_C_*, *Y_C_*, *Z_C_*) in the depth camera coordinate system, the transformation relationship can be described by Equation (1):

(1) $$\left[\begin{array}{c} X_{C}\\ Y_{C}\\ Z_{C} \end{array}\right]=R\left[\begin{array}{c} X_{L}\\ Y_{L}\\ Z_{L} \end{array}\right]+\left[\begin{array}{c} t_{l}\\ t_{2}\\ t_{3} \end{array}\right]=\left[\begin{array}{cc} R_{LtoC} & T_{LtoC}\\ 0 & 1 \end{array}\right]\left[\begin{array}{c} X_{L}\\ Y_{L}\\ Z_{L}\\ 1 \end{array}\right]$$

where $\left[R_{LtoC},T_{LtoC};0,1\right]$ is the external parameter matrix of the depth camera; ***R_LtoC_*** is the rotation matrix from the LiDAR coordinate system to the depth camera coordinate system; and ***T_LtoC_*** is the translation matrix from the LiDAR coordinate system to the depth camera coordinate system. Assuming the physical point has 2D coordinates (*u*, *v*) in the pixel coordinate system of the depth camera, the conversion relationship from 2D coordinates to 3D coordinates can be described by Equation (2):

(2) $$Z_{C}\left[\begin{array}{c} u\\ v\\ 1 \end{array}\right]=\left[\begin{array}{ccc} \frac{1}{d_{x}} & 0 & u_{0}\\ 0 & \frac{1}{d_{y}} & v_{0}\\ 0 & 0 & 1 \end{array}\right]\left[\begin{array}{cccc} f & 0 & 0 & 0\\ 0 & f & 0 & 0\\ 0 & 0 & 1 & 0 \end{array}\right]\left[\begin{array}{cc} R_{LtoC} & T_{LtoC}\\ 0 & 1 \end{array}\right]\left[\begin{array}{c} X_{L}\\ Y_{L}\\ Z_{L}\\ 1 \end{array}\right]$$

where *f* represents the focal length of the depth camera; *d_x_* and *d_y_* are the pixel-to-millimeter conversion factors along the *x*-axis and *y*-axis, respectively; and *u*_0_ and *v*_0_ define the offsets of the projection screen center relative to the optical axis. Thus, the correspondence problem between a physical point (*X_L_*, *Y_L_*, *Z_L_*) in the LiDAR coordinate system and a pixel point (*u*, *v*) in the depth camera pixel coordinate system can be transformed into a parameter solving problem.

The field of vision (FOV) refers to the range of view that the sensor can cover. Mechanical LiDAR typically has a 360° scanning range, whereas the depth camera has a smaller range. Therefore, before fusion, it is necessary to match the FOVs of the two sensors. This process involves removing the redundant point cloud data that LiDAR cannot cover, thereby reducing data redundancy and improving real-time performance in the fusion process [18].

### 2.2. Leaf Clustering Principle Based on Equidistantly Expanded Polygons

When identifying leaves, the YOLOv8 model is first used to obtain the leaf segmentation mask. In the field of computer vision, YOLOv8, as a state-of-the-art (SOTA) model, is built upon the YOLO series and incorporates a new backbone network architecture. It replaces the C3 module from YOLOv5 with the C2f module, achieving further lightweighting and enhancing feature extraction and processing capabilities. YOLOv8 also adopts a new anchor-free detection head, which no longer relies on anchor boxes, thereby improving adaptability to various target shapes and sizes. Additionally, YOLOv8 introduces a new loss function that enhances the model’s convergence speed and performance [19].

**Figure 2:**
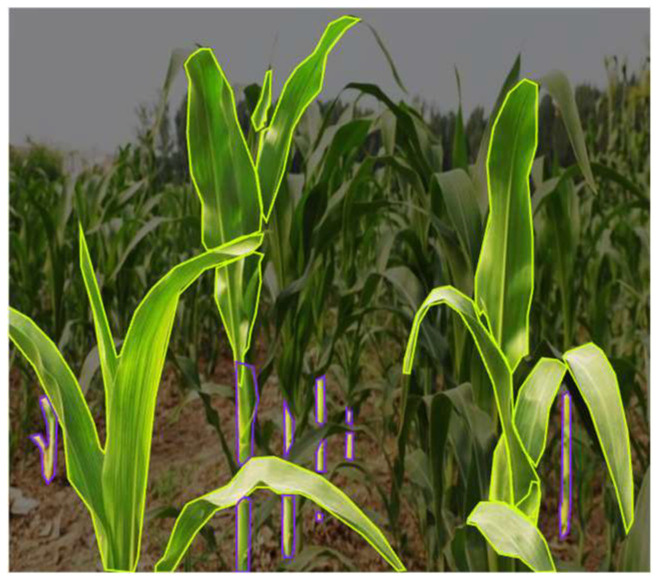
Annotations of Corn Leaves and Stalks in the YOLOv8 Model Training Dataset. Green outline indicates corn leaves; Purple outline indicates corn stalks.

YOLOv8 training dataset mainly comes from the seed production field in Zhangye, Gansu and the Shangzhuang Experimental Station of China Agricultural University. The data is collected by taking pictures at different locations at a certain time interval using an industrial camera. To ensure the diversity of data samples, the samples include images of the near-ground end of the corn field with different lighting, different angles, and different periods. A total of 1308 clear and available images of the near-ground end of the corn are collected. In order to enrich the dataset and improve the generalization ability of the network model to features, the dataset is expanded to 8012 images by performing data enhancement processing such as translation, rotation, flipping, and cropping on the images. After manually annotating the dataset using the Roboflow tool, the training set, validation set, and test set were distinguished according to a certain ratio and trained using YOLOv8. The annotations are shown in the Figure 2, where the image in the green outline is a corn leaf and the image in the purple outline is a corn stalk.

After obtaining the leaf segmentation mask, the mask is corroded and expanded using the built-in algorithm of OpenCV to remove noise points. At the same time, the leaf contour needs to be fitted into a polygon, and the number of polygon edges is adjusted according to actual needs. Considering that the actual recognition process often causes inaccurate leaf recognition contours due to problems such as leaf growth morphology and position, strong changes in illumination, etc., specifically, due to the small number of features and small plane area at the curled part of the leaf, a leaf may be identified as multiple leaves, thereby affecting the distance measurement.

**Figure 3:**
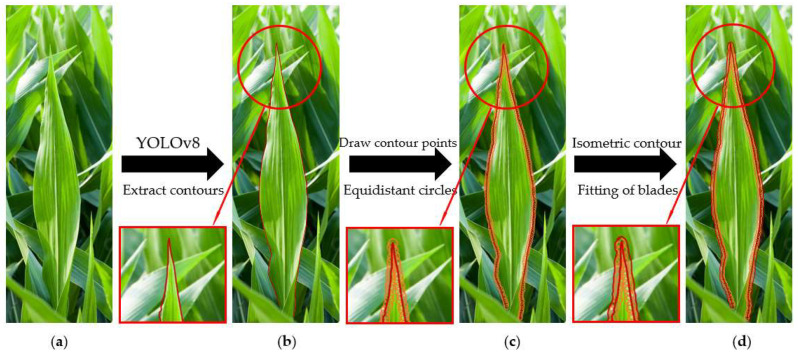
Process of equidistant contour generation: (**a**) actual shape of the leaf, (**b**) leaf Recognition results under YOLOv8, (**c**) drawing of equidistant circles around leaf contour points, (**d**) fitting of equidistant contours around leaf.

**Figure 4:**
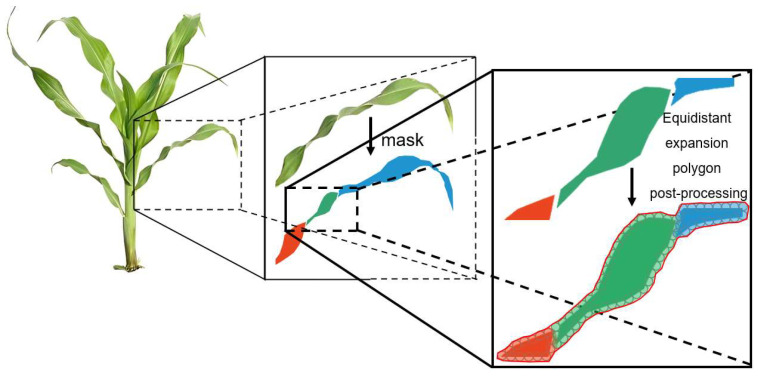
Post-processing results of leaf recognition using equidistant contour clustering. Red, green, and blue areas represent the identified discontinuous leaves. The red contour line shows the complete leaf outline after clustering using the equidistant expansion polygon post-processing algorithm.

To this end, each edge of the polygon fitted by the leaf is first divided into an appropriate number of n contour points by equal distance, and then a circle with a radius of equal distance value is drawn with each contour point as the center of the circle, and then the contours of n equidistant circles are fitted as equidistant contours. The equidistant contours are tangent to the equidistant circles, and the generation process is shown in Figure 3, where the number of contour points and the equidistant value need to be adjusted according to actual conditions. If the equidistant contours generated by the leaves overlap, the overlapping parts belonging to the same feature are clustered. The setting of the equidistant contour is mainly to solve the problem that the same leaf is identified as multiple discontinuous leaves due to curling and folding of corn leaves. The processing principle is that although the same leaf may be identified as discontinuous, the distance at the junction is small, and the distance between different leaves is large. Therefore, the equidistant contour is used to cluster the discontinuously identified leaves, thereby achieving complete leaf recognition. The post-processing effect is shown in Figure 4. For the stems, due to the occlusion of the leaves on the stems, it is difficult to cluster the equidistant contours. For this reason, the discontinuous identification of the stems is further processed below.

### 2.3. Distance Measurement Principle Based on Multi-Sensor Fusion

After jointly calibrating the extrinsics of the depth camera and LiDAR, the contours of corn leaves or stalks can be projected into a three-dimensional point cloud formed by the depth camera and LiDAR. This projection helps in removing points outside the contours to reduce computational load and enhance real-time processing. To mitigate the impact of invalid point clouds within the leaves or stalks contours on distance measurement accuracy, the Euclidean clustering algorithm is employed.

The fundamental principle of the Euclidean clustering algorithm is to determine if points belong to the same cluster based on their Euclidean distance from neighboring points [20]. Initially, for a point *p* in the point set *P* within the contour, the algorithm uses a tree data structure (kd-tree) to find *k* nearest neighbors and checks if the distance between these *k* neighbors and *p* is less than a threshold *r* [21]. If it is, these points are added to the cluster set *U*. The calculation formula for the Euclidean distance between two points is shown in Equation (3). This algorithm is suitable for scenarios where there is a significant difference in point cloud densities between different classes. Invalid point clouds, which fall outside the leaves or stalks, exhibit a larger distance, making the Euclidean clustering algorithm effective for removing invalid point clouds within the contours.

(3) $$\begin{array}{l} d(p_{i},p_{j})=\sqrt{{\left(x_{i}-x_{j}\right)}^{2}+{\left(y_{i}-y_{j}\right)}^{2}+{\left(z_{i}-z_{j}\right)}^{2}} \end{array}$$

where (*x_i_*, *y_i_*, *z_i_*) and (*x_j_*, *y_j_*, *z_j_*) are the three-dimensional coordinates of points *i* and *j* in the point set *P*. Secondly, for the remaining points in *U*, repeat the above steps sequentially. If no new points are added to *Q*, place *Q* into the list *C* and clear *Q* to await the next clustering iteration.

Due to the higher density of the point cloud generated by the depth camera compared to the LiDAR, it is necessary to perform sampling on the depth camera point cloud to reduce the computational data volume. Initially, a collection of small 3D voxel grids is created, followed by the calculation of the centroid for each 3D voxel. Given that the centroid point may not correspond to an actual point within the original point cloud, a k-nearest neighbor search is employed to identify the original point nearest to the centroid. This nearest original point is then utilized in place of the centroid to preserve the fine details of the point cloud. Thus, the final depth camera point cloud set is constructed. Let $U=\left\{\begin{array}{c} \vec{u_{1}},\cdots \vec{u_{n}} \end{array}\right\}$ denote the point cloud of the leafs or stalks obtained through sampling by either the LiDAR or depth camera. The probability density function of $U=\left\{\begin{array}{c} \vec{u_{1}},\cdots \vec{u_{n}} \end{array}\right\}$ is defined as follows:

(4) $$p(\vec{u_{k}})=\frac{1}{{\left(2\pi \right)}^{3/2}\sqrt{\left|C\right|}}\exp(-\frac{{\left(\vec{u_{k}}-\vec{q}\right)}^{T}C^{-1}(\vec{u_{k}}-\vec{q})}{2})$$

where $\vec{q}$ and **C** respectively represent the mean and covariance matrix of the cube containing the points:

(5) $$\vec{q}=\frac{1}{n}\sum_{k}\vec{u_{k}}$$

(6) $$C=\frac{1}{n-1}\sum_{k}\left(\vec{u_{k}}-\vec{q}\right){\left(\vec{u_{k}}-\vec{q}\right)}^{T}$$

Using $T(\vec{p},\vec{u_{k}})$ to denote the spatial transformation relationship between two frames of point clouds, we have:

(7) $$\vec{p}={\left[t_{x},t_{y},t_{z},\phi_{x},\phi_{y},\phi_{z}\right]}^{T}$$

(8) $$T(\vec{p},\vec{u_{k}})=\left[\begin{array}{ccc} c_{y}c_{z} & -c_{y}s_{z} & s_{y}\\ c_{x}s_{z}+s_{x}s_{y}c_{z} & c_{x}c_{z}-s_{x}s_{y}c_{z} & -s_{x}c_{y}\\ s_{x}s_{z}-c_{x}s_{y}c_{z} & c_{x}s_{y}s_{z}+s_{x}c_{z} & c_{x}c_{y} \end{array}\right]\vec{u_{k}}+\left[\begin{array}{c} t_{x}\\ t_{y}\\ t_{z} \end{array}\right]$$

where $t_{x},\;t_{y},\;t_{z},\;\phi_{x},\;\phi_{y},\;\phi_{z}$ represent the spatial translation and rotation along three axes, with $c_{i}$ denoting $\cos\phi_{i}$ and $s_{i}$ denoting $\sin\phi_{i}$. To solve for the pose transformation of the current point cloud when the likelihood function is maximized, i.e., when $T(\vec{p},\vec{u_{k}})$ is most similar to the reference point cloud $\vec{p}$, $s(\vec{p})$ is defined as a function to measure similarity before and after transformation:

(9) $$s(\vec{p})=-\sum_{k=1}^{n}p(T(\vec{p},\vec{u_{k}}))$$

The Levenberg–Marquardt (LM) algorithm, also known as Damped Least–Squares, is employed to find the minimum of $s(\vec{p})$ function. It combines the advantages of gradient descent and Gauss–Newton methods. In the LM algorithm, a trust region radius is introduced to adjust the algorithm’s characteristics: when small, it computes the optimal step length similar to the Gauss–Newton method; when large, it resembles gradient descent’s optimal step length formula. Dynamically adjusting the step length for each iteration by changing the trust region radius ensures good approximation with each Taylor expansion, achieving local convergence. This approach prevents issues such as failure to converge or being trapped in local minima due to excessively large step sizes. The equation to solve is:

(10) $$(H+\lambda I)\Delta \vec{p}=-\vec{g}$$

where H and $\vec{g}$ represent the Hessian matrix and gradient vector of function $s(\vec{p})$, respectively, and are defined as:

(11) $${\vec{g}}_{i}=\frac{\partial s}{\partial p_{i}}=\sum_{k=1}^{n}{\vec{u}}_{k}^{-T}C^{-1}\frac{\partial {\vec{u}}_{k}^{-}}{\partial p_{i}}\exp(-\frac{{\vec{u}}_{k}^{-T}C^{-1}{\vec{u}}_{k}^{-}}{2})$$

(12) $$H_{ij}=\frac{\partial^{2}s}{\partial p_{i}p_{j}}=\sum_{k=1}^{n}\exp(-\frac{{\vec{u}}_{k}^{-T}C^{-1}{\vec{u}}_{k}^{-}}{2})\left[-({\vec{u}}_{k}^{-T}C^{-1}\frac{\partial {\vec{u}}_{k}^{-}}{\partial p_{i}})({\vec{u}}_{k}^{-T}C^{-1}\frac{\partial {\vec{u}}_{k}^{-}}{\partial p_{j}})+{\vec{u}}_{k}^{-T}C^{-1}\frac{\partial^{2}{\vec{u}}_{k}^{-}}{\partial p_{i}\partial p_{j}}+\frac{\partial {\vec{u}}_{k}^{-}}{\partial p_{j}}C^{-1}\frac{\partial {\vec{u}}_{k}^{-}}{\partial p_{i}}\right]$$

λI is a positive definite diagonal matrix, where λ represents the trust region radius with λ ≥ 0. During each iteration, the LM algorithm calculates the factor ρ to assess the accuracy of the Taylor approximation. It dynamically adjusts the size of λ based on ρ. The formula for calculating ρ is as follows:

(13) $$\rho =\frac{s(\vec{p}+\Delta \vec{p})-s(\vec{p})}{J{\left(\vec{p}\right)}^{T}\Delta \vec{p}}$$

Here, $\Delta \vec{p}$ represents the increment of the pose in each iteration of the $s(\vec{p})$ function. When ρ ≤ 0.25, the Taylor approximation is considered poor, requiring a reduction in λ, where $\lambda_{k+1}=0.5\lambda$ is chosen. When ρ ≥ 0.75, the Taylor approximation is deemed accurate, necessitating an increase in λ, where $\lambda_{k+1}=2\lambda$ is chosen. When 0.25 ≤ ρ ≤ 0.75, λ is maintained at its current value.

Once the convergence condition of the LM method is met, the pose transformation between the two point clouds $\vec{p}$ can be determined. Considering that the data return frequency of depth camera and LiDAR is much lower than that of IMUs, and that the time interval between the two frames is stabilized at the millisecond level, direct integration of acceleration would result in rapid error accumulation. Therefore, only the three-axis angular velocity measured by the IMU is directly integrated between the timestamps of the two point clouds, as shown in the equation.

(14) $$\phi {’}_{x}=\int_{t}^{t+\Delta t}\omega_{x}dt$$

(15) $$\phi {’}_{y}=\int_{t}^{t+\Delta t}\omega_{y}dt$$

(16) $$\phi {’}_{z}=\int_{t}^{t+\Delta t}\omega_{z}dt$$

Define the rotational variables between every two frames of data from the depth camera and LiDAR as $\phi_{D\_x}$, $\phi_{D\_y}$, $\phi_{D\_z}$ and $\phi_{L\_x}$, $\phi_{L\_y}$, $\phi_{L\_z}$, respectively. To calculate the difference matrices $\Delta \phi_{D}$ and $\Delta \phi_{L}$ between the rotation matrices of the depth camera and LiDAR point cloud data relative to the IMU rotation matrix, we have:

(17) $$\Delta \phi_{D}=[\phi_{D\_x}-\phi {’}_{x},\phi_{D\_y}-\phi {’}_{y},\phi_{D\_z}-\phi {’}_{z}]$$

(18) $$\Delta \phi_{L}=[\phi_{L\_x}-\phi {’}_{x},\phi_{L\_y}-\phi {’}_{y},\phi_{L\_z}-\phi {’}_{z}]$$

Then, the confidence factor k for the point clouds from the two types of sensors can be obtained by the following equation:

(19) $$k=\frac{\left||\Delta \phi_{L}|\right|}{\left||\Delta \phi_{L}||+||\Delta \phi_{D}|\right|}$$

Define the depth camera point cloud set as $U_{D}$ and the LiDAR point cloud set as $U_{L}$. The fused point cloud, which is the optimal confidence point cloud, $C_{conf}$, is given by:

(20) $$C_{conf}=kU_{L}+(1-k)U_{D}$$

The optimal confidence point cloud performs point cloud matching for the data obtained from the two types of sensors, and then evaluates their accuracy based on IMU data. The two point cloud sets are fused according to the confidence factor. This approach avoids the problem of reduced accuracy in point cloud data from a single sensor due to significant environmental changes during the robot’s field operations, thereby improving distance measurement accuracy.

### 2.4. Stem FILL-DBSCAN Clustering Algorithm Based on Projection

DBSCAN is a point cloud clustering algorithm designed for datasets with significant density features [22]. Given that point clouds belonging to the same stem exhibit higher density characteristics, this algorithm can be effectively utilized for clustering stem point clouds. During the DBSCAN clustering process, the neighborhood distance threshold is set to 0.01 m, and the minimum number of points in the core point’s neighborhood is set to 20. To enhance clustering efficiency, the KD-Tree (K-Dimensional Tree) search algorithm is incorporated into the DBSCAN algorithm to accelerate the search for neighboring points.

**Figure 5:**
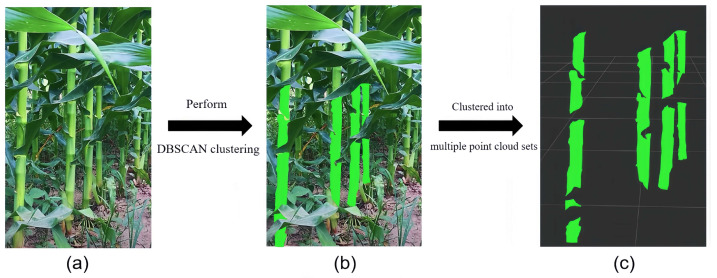
Misidentification of stems under leaf occlusion. (**a**) Real scene of corn stalk testing. (**b**) DBSCAN clustering of detected stalk occlusion point clouds. (**c**) Due to leaf overlap, a single corn stalk is clustered into multiple point cloud sets.

**Figure 6:**
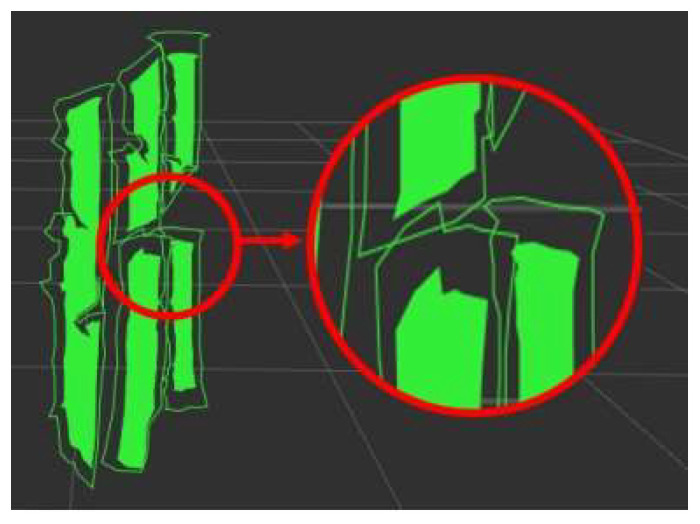
Post-processing of stem with equidistant outward-expanded polygon.

Typically, after performing DBSCAN clustering on all optimal confidence point clouds within the detected stem mask region, numerical calculations can be carried out to complete the distance measurement of the stem. However, due to the cross-occlusion between leaves, a single corn stem may be clustered into multiple point cloud sets, as shown in Figure 5, which increases the number of variables and may even affect the subsequent statistics of effective stems. Unlike the discontinuous recognition issues caused by factors such as the shape and lighting of leaves, different point cloud sets of the same stem are significantly distant from each other. Using equidistant outward-expanded polygons for post-processing stem point clouds requires increasing the equidistant value, which may result in different stems being incorrectly identified as the same stem, as shown in Figure 6.

**Figure 7:**
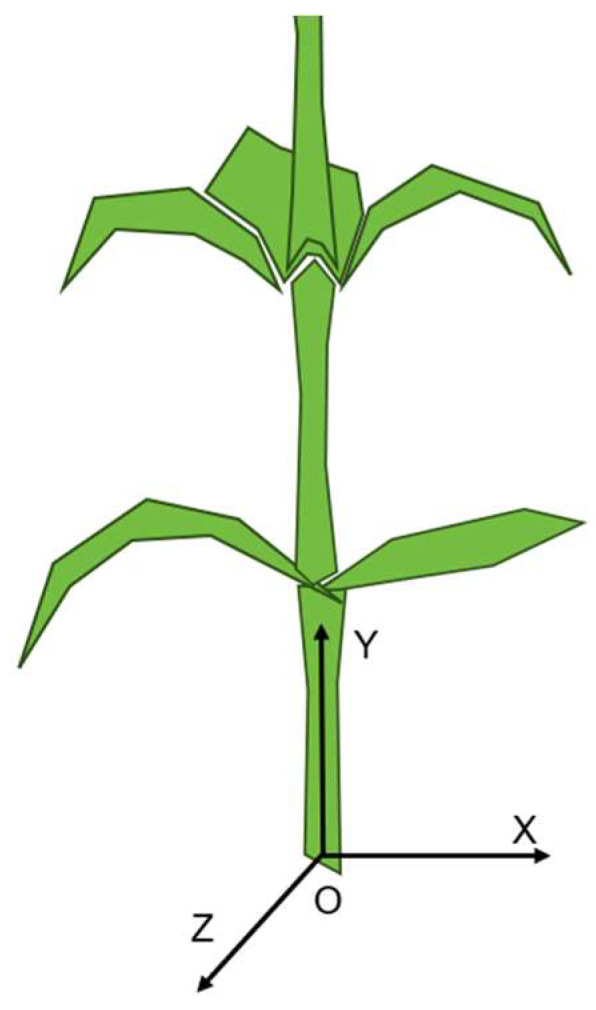
Definition of the XYZ directions for the corn stalk.

Considering that multiple point cloud sets of the same stalk have a large distance difference only in the Y direction but still belong to the same stalk after being projected to the X-Z plane, the definition of the XYZ direction is shown in the Figure 7. Therefore, a density-based dimensionality reduction clustering FILL-DBSCAN algorithm is proposed to accurately cluster multiple point clouds belonging to the same stalk. First, the optimal confidence point cloud of the stalk is projected onto the X-Z plane, and the point cloud on the 2D plane is clustered by DBSCAN. After determining the stalk segments belonging to the same plant, the point cloud is clustered in three-dimensional space, thereby realizing accurate identification and distance measurement of corn stalks of the same plant. In actual corn planting, the row spacing is maintained at more than 50 cm, the plant spacing is usually around 40 cm, and when the robot is running in the corn field, it can only scan the features near the ground end of the corn stalks. Therefore, the FILL-DBSCAN algorithm can still achieve effective clustering when the stalk has a certain inclination.

## 3. Experiment

### 3.1. Experiment on Equidistant Outward-Expanded Polygon Post-Processing Algorithm

The effectiveness of the fusion recognition and ranging algorithm depends on the recognition enhancement of the equidistant expansion polygon of the leaves, the ranging accuracy of the optimal confidence point cloud, and the ranging enhancement of the 2D projection clustering of the stems. This section uses a corn physical model to verify the recognition enhancement of the equidistant expansion polygon. First, the YOLOv8 model needs to be used to train the corn leaves and stalks recognition model to recognize corn leaves and stalks in real time.

**Figure 8:**
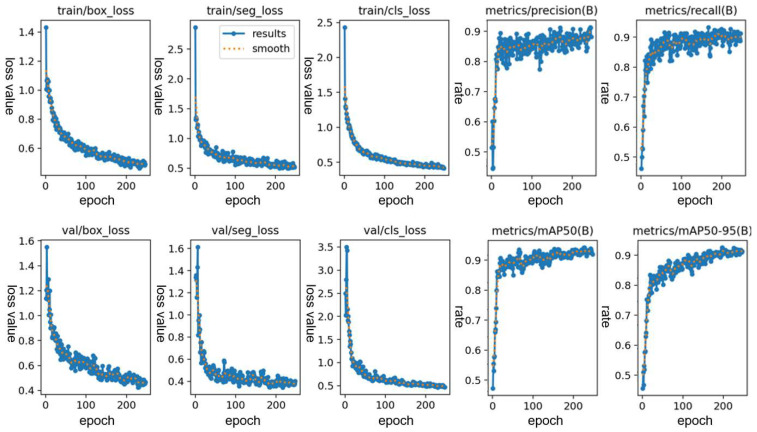
Training results of YOLOv8l model.

During training, the YOLOv8l model is used. This model is a larger model in the YOLO series, with relatively high accuracy but relatively low detection speed. Since the actual field operation speed of the emasculating robot is low and the industrial computer is equipped with a GPU, it is suitable to use the l model for more accurate training. The appropriate setting of training parameters has a great impact on the recognition accuracy of the subsequent model. After adjustment, epochs is set to 300 and batch is set to 8. The training results are shown in the Figure 8. The model accuracy, recall rate and average precision are 92.1%, 91.4%, and 94.9%, respectively. It is practical and can support the effective recognition of corn leaves and stalks.

**Figure 9:**
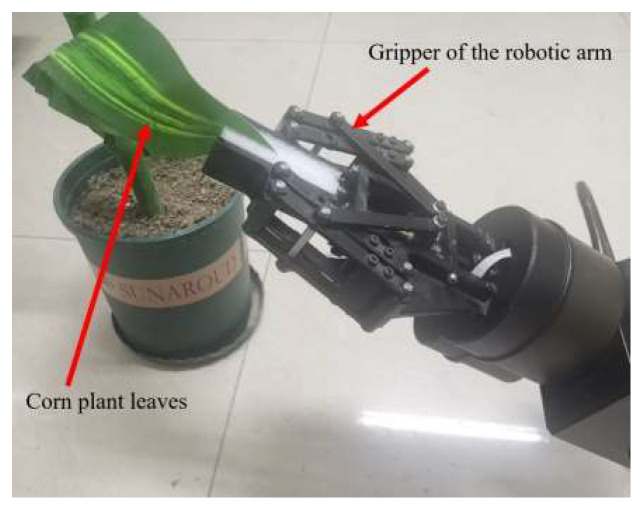
Simulation of curved leaf morphology.

To simulate different growth positions and characteristics of real corn leaves in a depth camera, we use a robotic arm to grasp the tip of a leaf, as shown in Figure 9. The robotic arm’s gripper faces the base of the leaf, and its initial position depends on the natural initial position of the tip of the leaf. The gripper is controlled to rotate around the leaf’s main vein by 15° per rotation and to translate radially along the stem by 1 cm per movement.

**Figure 10:**
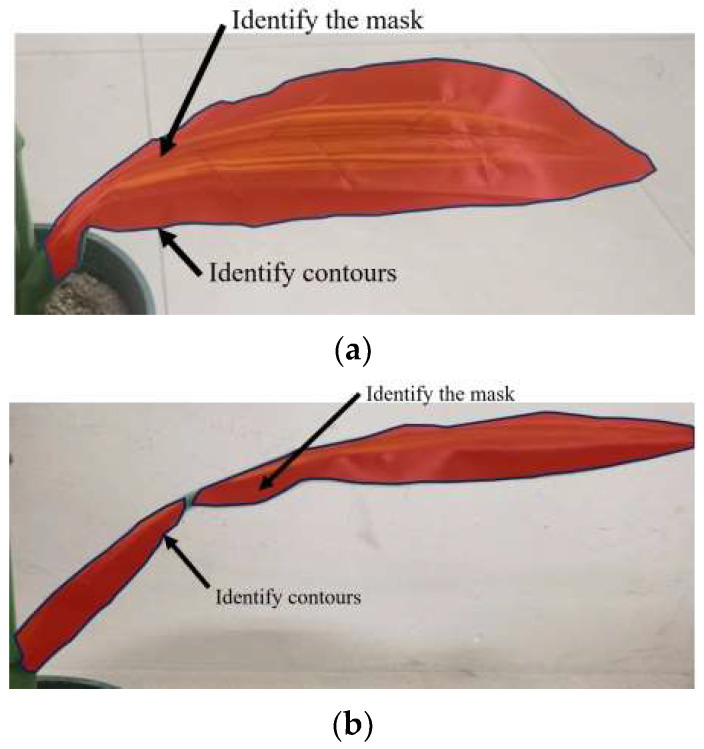

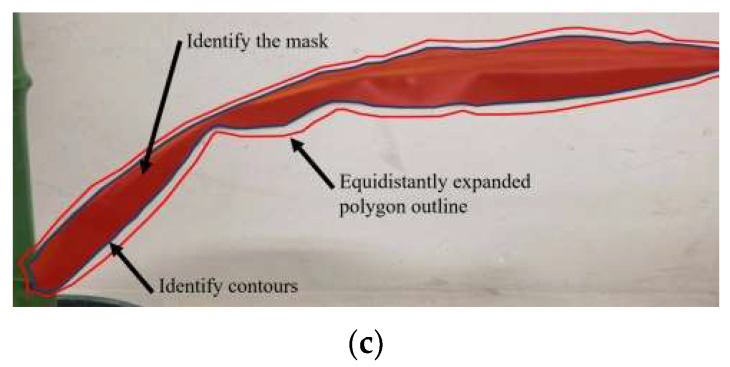
Test of equidistant expansion polygon post-processing algorithm: (**a**) Recognition of uniform leaf morphology. (**b**) Recognition of curved leaf morphology. (**c**) Result of leaf post-processing with equidistant outward-expanded polygons.

**Figure 11:**
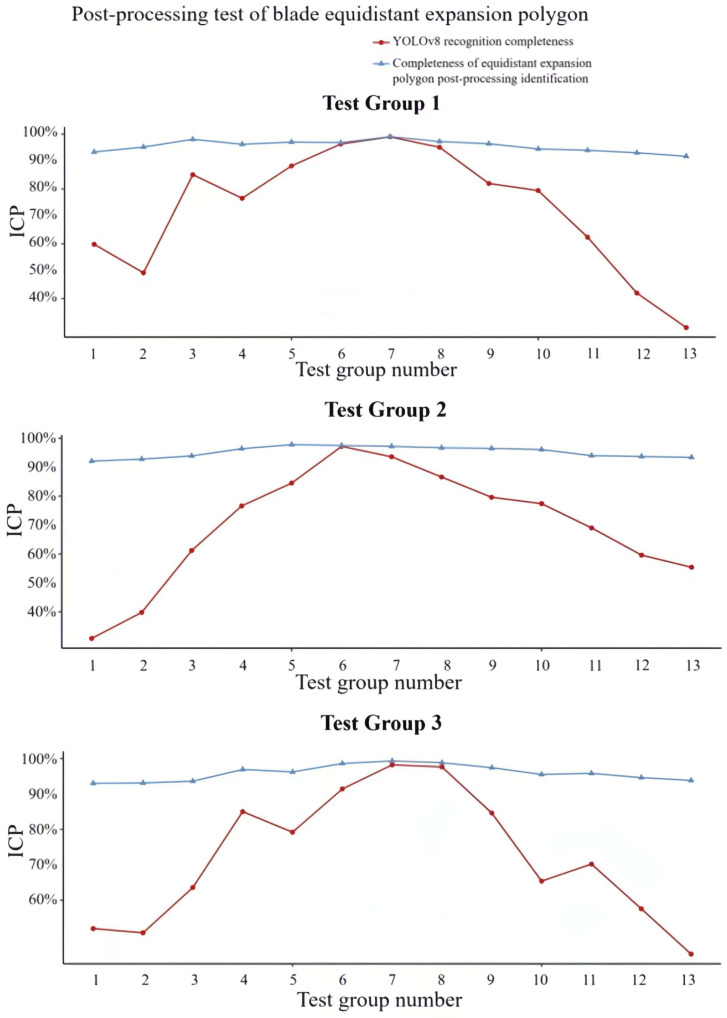
Test of leaf post-processing with equidistant outward-expanded polygons. ICP: identification completeness percentage.

When the shape of the leaves in the image is relatively uniform and the change of the projection area in the X and Y directions is relatively gentle, YOLOv8 can obtain a high recognition rate, as shown in Figure 10a. However, when the projection area in the X and Y directions changes greatly, that is, when the leaves are curved, the recognition rate of YOLOv8 will decrease, and a single leaf may be mistaken for multiple leaves, as shown in Figure 10b. The use of equidistant polygon expansion for post-processing will cause the contours to overlap, and the overlapping parts of the contours can be used to cluster the same leaf, as shown in Figure 10c. According to the above post-processing principle, three groups of tests were set up using three leaves, and the rotation angle and translation distance of the manipulator gripper of each test group are shown in Table 1. The results of the three groups of tests are shown in Figure 11, where the recognition completeness value of YOLOv8 and the recognition completeness value of the equidistant expansion polygon post-processing are expressed as identification completeness percentage (ICP). ICP can be expressed by the proportion of frames with complete leaf recognition in the 500 frames before and after the current moment. In the three groups of tests, the highest recognition completeness reached 95.89%, and the average recognition completeness was 95.61%. It can be seen that the equidistant polygon expansion post-processing algorithm enhances the integrity of the recognition results and plays a vital role in improving the measurement accuracy.

When corn leaves are relatively sparse, meaning the leaves do not overlap, the equidistant expansion polygon post-processing algorithm effectively re-identifies discontinuous leaf segments caused by factors such as lighting or curling as a single leaf. However, when the corn leaves are dense and overlapping occurs, this algorithm may mistakenly cluster overlapping leaves in the 2D projection as a single leaf. To address this issue, depth camera point cloud data is utilized, and the Euclidean distance clustering algorithm is applied to determine whether the point clouds within the leaf contour generated by the post-processing algorithm belong to the same leaf. If the point clouds within the contour meet the Euclidean distance clustering criteria, meaning that the variation in distance between adjacent point clouds is within a certain range, the point clouds can be considered to belong to the same leaf, indicating that the contour encloses a single leaf. Conversely, if a sufficient number of point clouds exhibit a sudden change in distance, failing to meet the Euclidean distance clustering criteria, it can be determined that the point clouds within the contour generated by the post-processing algorithm do not belong to the same leaf. In such cases, the non-primary point clouds and the feature points of the contour polygon are removed, retaining only the contour of the primary identified leaf.

### 3.2. Experiment on Optimal Confidence Point Cloud Distance Measurement

**Figure 12:**
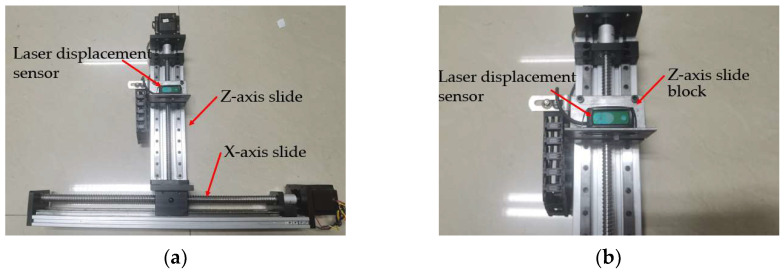
Laser displacement sensor test platform.

Since the ranging accuracy of LiDAR and depth camera is stable at centimeter level, while the accuracy of laser displacement sensor is stable at millimeter level, we first use a BLF-200NM-485 laser displacement sensor to measure the relative distance between corn leaves and stalks features. The sensor has a detection range of 100 mm–2000 mm, a resolution of 1 mm, and a ranging accuracy of 2 mm, which is higher than the ranging accuracy of LiDAR and depth camera. Considering that the laser displacement sensor can only perform high-precision measurements of single-point distances, a dual-axis X-Z slide table was constructed as shown in Figure 12a,b. The laser displacement sensor was fixed on the slider of the *Z*-axis slide table, enabling high-precision measurements of discrete curved surfaces within the plane.

**Figure 13:**
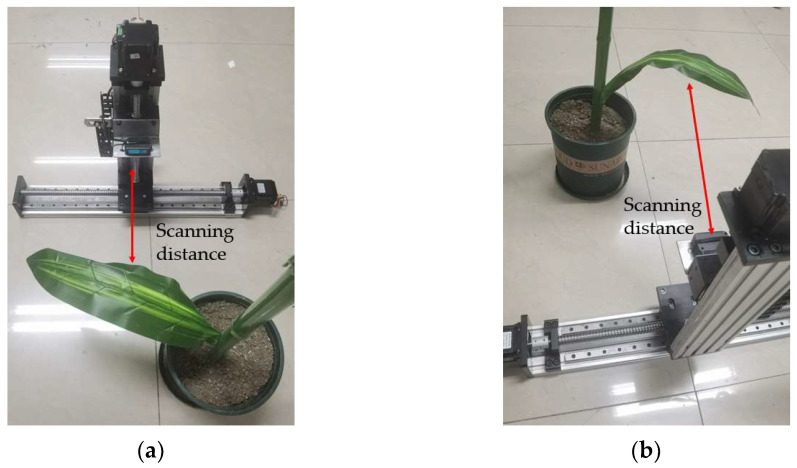
Laser displacement sensor measurement test setup: (**a**) Frontal scanning measurement; (**b**) Rear scanning measurement.

**Figure 14:**
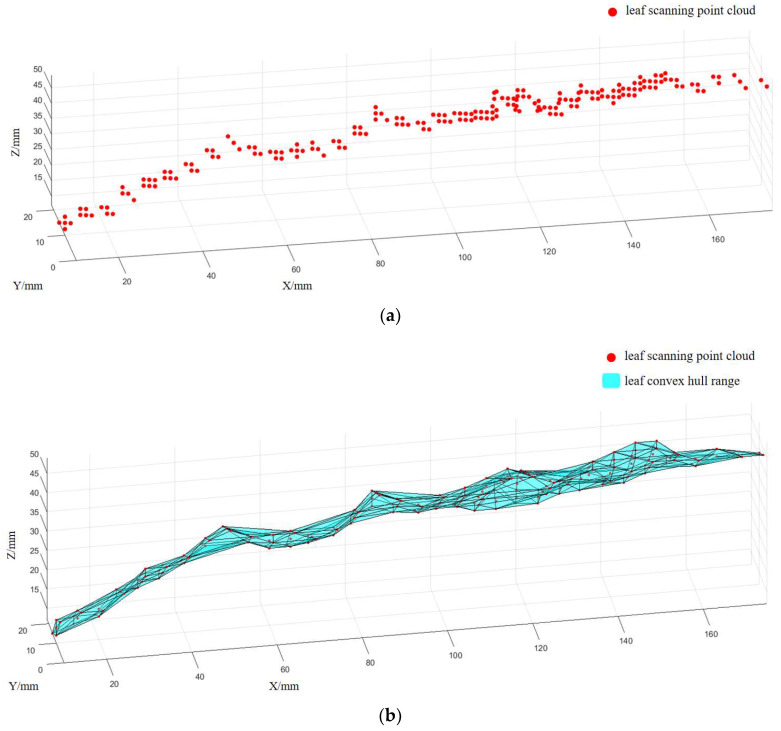
Leaf scanning results: (**a**) distance point cloud obtained from scanning; (**b**) leaf convex hull shape extracted using the α-shape algorithm.

Using the corn model for experimentation, as shown in Figure 13, the setup involves controlling *X*- and *Z*-axis slide table motors via RS485. The *X*-axis motor drives the screw and slider at a speed of 5 mm/s. When reaching the limit position or zero position of the *Z*-axis slide table, the *Z*-axis motor moves the slider upward by 2 mm, enabling discrete scanning with the laser displacement sensor. After scanning, a set of points including corn features is obtained. Points outside the corn features are filtered out for downsampling, and the point cloud data is imported into MATLAB to obtain results as shown in Figure 14a, primarily illustrating the scan results of the leaves. Due to significant variations in leaf morphology, individual distance points cannot fully reflect the overall characteristics of the corn leaves. Therefore, the mean of effective distance points in the depth direction is used as the distance value of the leaves, and the α-shape is employed to describe the leaf shape, as depicted in Figure 14b.

The α-shape algorithm is used to extract convex hull shapes from point cloud data [23]. Based on the convex hull generation method of Delaunay triangulation, this algorithm effectively extracts shapes with convex hull characteristics from discrete point cloud data. The basic idea of the α-shape algorithm is to control a parameter α to determine the shape of the convex hull. Different values of α yield different shapes of the convex hull. Specifically, when α is small, the shape of the convex hull closely resembles the original point cloud data, whereas a larger α results in a smoother shape of the convex hull [24].

In the α-shape algorithm, Delaunay triangulation is first performed. Delaunay triangulation divides a set of points into non-overlapping triangles and has favorable properties for describing adjacency relationships between points. After obtaining the Delaunay triangulation, the convex hull boundary can be filtered based on boundary conditions. For each edge, if there exists a circle such that both endpoints of the edge and all points on the circle are within the α radius, then the edge belongs to the convex hull boundary.

There are libraries in MATLAB and the Point Cloud Library (PCL) that implement the α-shape algorithm, facilitating its fast invocation across different platforms. After obtaining the convex hull shape of the leaves, the leaves are projected onto a plane perpendicular to the line connecting the maize axis and the robot axis. The extreme points of the projected leaves in the vertical and horizontal directions relative to this plane are collected as feature points Q for subsequent tests.

**Figure 15:**
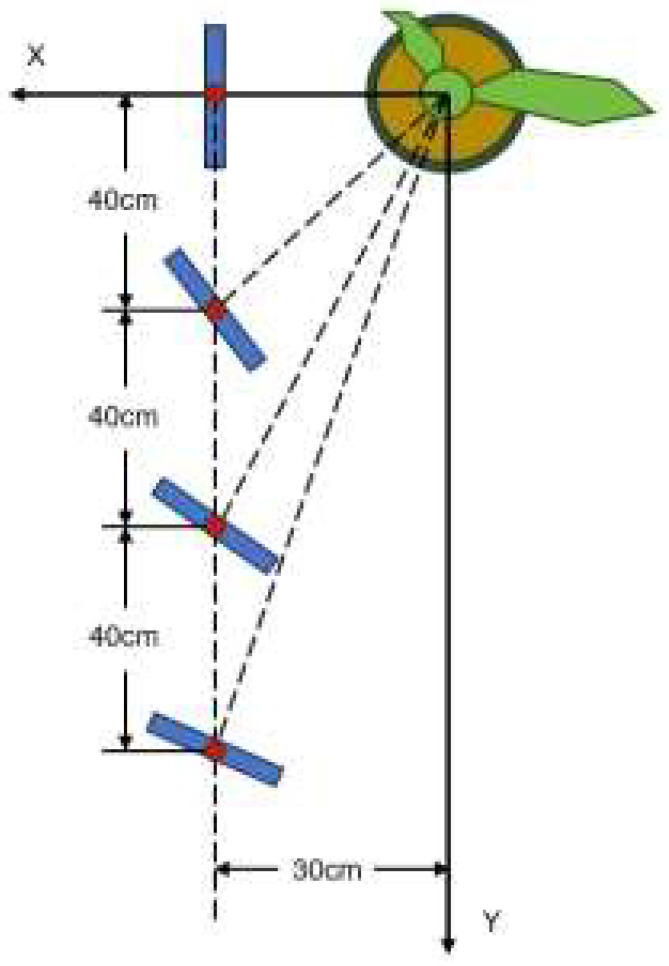
Measurement point setup.

Based on the above data processing workflow, to further test the effectiveness of the algorithm when the robot operates in a maize field, a plane Cartesian coordinate system is established with the maize simulation model as the origin. Considering that the typical row spacing in real maize fields is often 60 cm, four measurement points are set at 30 cm horizontal distance from the maize, spaced 40 cm apart, as shown in Figure 15. Using the laser displacement sensor, relative distances of maize leaf features are measured. The measurement results are presented in Table 2.

**Figure 16:**
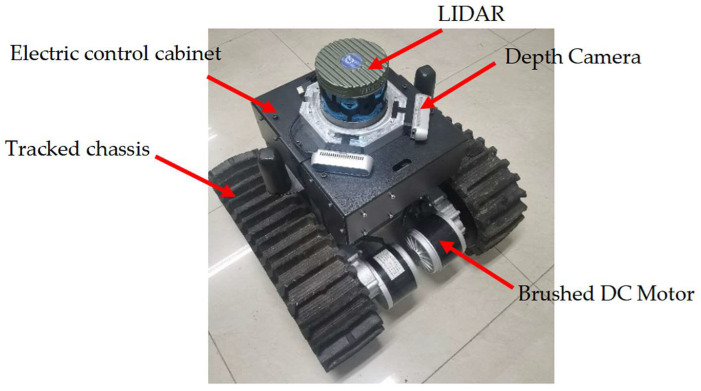
Experimental prototype.

Using the laser displacement sensor, accurate distances of maize leaves relative to the measurement points can be measured. To quantitatively analyze the actual ranging accuracy of the optimal confidence point cloud, an experimental prototype was constructed as shown in Figure 16. The prototype has dimensions of 48 cm in length, 45 cm in width, and 42 cm in height. It utilizes a tracked chassis as the motion unit, with DC brushed motors driving the tracks on both sides. After reduction by a gear reducer, the maximum travel speed is 0.3 m/s, and the minimum turning radius is 24 cm. The chassis motor driver, lithium battery, and transformer are placed on top of the robot chassis. The industrial computer, GNSS module, IMU, UWB positioning module, etc., are housed in the electronic control cabinet above the robot. The TM16 LiDAR is placed above the electric control cabinet. It is a 16-line multi-line LiDAR with a measurement range of 0.2~150 m. The ranging accuracy is ±5 cm within the range of 0.2–0.5 m, and the ranging accuracy is ±2 cm above 0.5 m. The RealsenseD435 depth camera is above the electric control cabinet and below the LiDAR and is arranged symmetrically. The ideal ranging range of the depth camera is 0.3~3 m, the depth field of view is 87° × 58°, and the ranging error within 2 m is less than 2%. Under the feedback of UWB, the chassis is controlled to move at a speed of 0.1 m/s along the dashed line in the diagram, and distance values obtained by the optimal confidence point cloud measurement are recorded at four measurement points.

**Figure 17:**
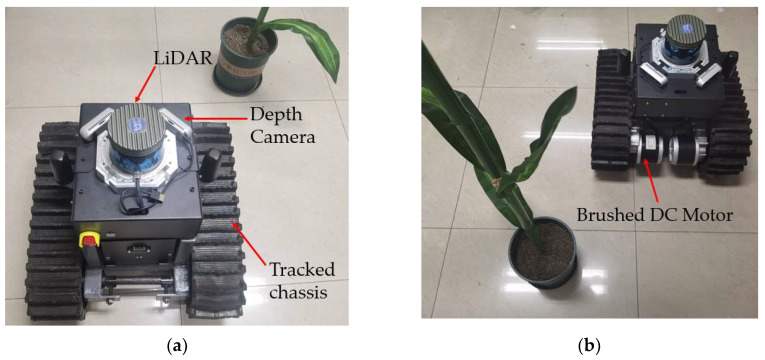
Upper interface of prototype test: (**a**) shows the physical maize leaves and recognition results, (**b**) displays the distance measurement results from the depth camera and LiDAR point clouds.

**Figure 18:**
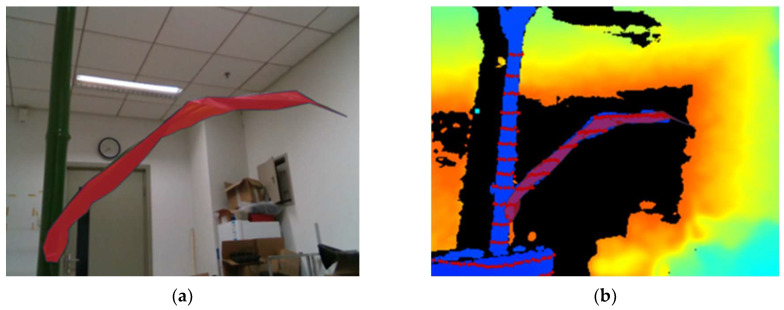
Interface of the prototype test on the host computer: (**a**) Real corn leaf and recognition results. The blue outline represents the identified contours, and the red area indicates the identified mask. (**b**) Depth camera and LiDAR point cloud measurement results.

Distance values collected from point clouds on the leaves are converted into projection distances to obtain the point cloud average and the extreme values of the four convex hulls. The experimental setup is depicted in Figure 17, and the measurement scenario is shown in Figure 18. The experimental results are presented in Table 3.

Comparing Table 2 and Table 3 reveals that the distance accuracy of the optimal confidence point cloud is lower when the distance is less than 30 cm, with an average distance measurement error of 3.1 cm and a maximum error of 3.4 cm for convex hull feature points. Beyond 30 cm, there is a significant improvement in distance accuracy, with an average measurement error of 0.51 cm and a maximum error of 0.25 cm. In order to reflect the accuracy of the optimal confidence point cloud algorithm in ranging, a single sensor was used for measurement and comparison. If only the LiDAR was used, the mean ranging error was 3.5 cm and the maximum error of the convex hull feature point was 6.9 cm. If only the depth camera was used, the mean ranging error was 4.1 cm and the maximum error of the convex hull feature point was 4.7 cm. It can be seen that the ranging accuracy of the optimal confidence point cloud is due to a single sensor, which verifies the effectiveness of the multi-sensor fusion solution.

The primary reason for the lower accuracy within 30 cm is due to the measurement characteristics of the sensors, where both the depth camera and LiDAR experience a sharp decrease in measurement precision. Therefore, even with multi-sensor fusion, distance measurement accuracy is challenging to guarantee. Conversely, beyond 30 cm, sensor distance measurement performance is normal, resulting in notably improved accuracy.

For the convex hull feature points at the same measurement location, due to the large scanning measurement graduation value of the BLF-200NM-485 laser displacement sensor, edge points may be missed, and the depth camera and LiDAR have poor edge segmentation capabilities, which often lead to point cloud jitter at the edge position. At the same time, due to the interference of natural wind, the sensor’s edge detection value may also jitter. Therefore, there is a certain error in the convex hull feature points, but for corn field navigation operations, the centimeter-level error is still within the allowable range.

By utilizing a high-precision laser displacement sensor to scan and measure corn features, particularly leaves, high-accuracy distance measurements are obtained. This validates the effectiveness of the proposed fusion algorithm in distance measurement tests at the same test points.

### 3.3. FILL-DBSCAN Post-Processing Algorithm Test

The post-processing algorithm using equidistant expansion polygons addresses the issue of misidentification of leaves during recognition due to leaf shape and lighting variations. It utilizes features close in distance to the mask after misidentification, employing equidistant expansion polygons to connect and cluster multiple masks of a single misidentified leaf, thereby achieving accurate leaf recognition. Unlike leaves, maize stalks are often misidentified as multiple masks due to leaf obstruction, resulting in greater distances between these masks within the same maize plant. Consequently, equidistant expansion polygons cannot effectively connect these segments. However, post-misidentification, the number of stalks significantly increases, complicating navigation operations. Therefore, it is essential to utilize the FILL-DBSCAN post-processing algorithm to cluster multiple misidentified stalk masks.

**Figure 19:**
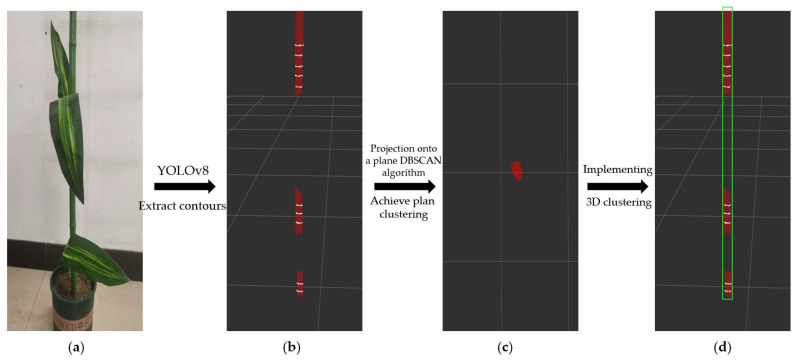
Stalk FILL-DBSCAN test: (**a**) corn model, (**b**) segmented corn stems identified by YOLOv8, (**c**) plane clustering using DBSCAN algorithm after point cloud projection, (**d**) three-dimensional clustering of segmented point clouds of the same corn stem.

Simulating the scenario where maize stalks are obstructed using a maize model, as shown in Figure 19a, the stalk is divided into three segments. After recognition by YOLOv8, these segments are similarly identified as three segments, with substantial distances between the point clouds of these segments, as depicted in Figure 19b. Projecting these three segments onto a plane and utilizing the DBSCAN algorithm achieves plane clustering, as shown in Figure 19c, thereby enabling three-dimensional clustering of multiple segments of stalk point clouds from the same maize plant, as shown in Figure 19d. The corn model was placed on a rotating platform and rotated in 5° increments. Clustering tests were conducted on the stalks at different angles. Out of 72 experimental groups, 69 successfully identified and completed clustering. In three groups, the clustering stability was low due to a large occlusion area and low identification accuracy. The FILL-DBSCAN post-processing algorithm achieved a 95.8% success rate in recognizing the same corn stalks under leaves occlusion, which is sufficient to meet actual navigation needs.

### 3.4. Corn Field Validation Test

**Figure 20:**
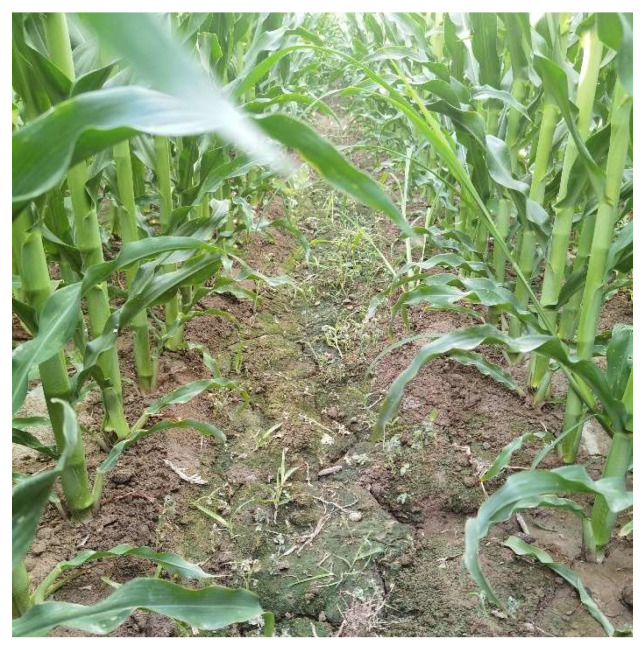
Actual conditions of the testing scenario in the corn field.

To further verify the effect of the algorithm in this paper on actual corn fields, a corn field in Zhangye, Gansu was selected for field testing. The actual test scene is shown in the Figure 20. Different from laboratory tests, the morphology of corn leaves in the natural state will change greatly within a day, and the natural wind will also affect the leaf morphology, resulting in obvious errors in the leaf measurement values before and after the test. The change in illumination will directly affect the recognition effect of YOLOv8 and the post-processing algorithm. Therefore, the test time needs to be strictly controlled to reduce the test error. A total of 500 corn leaves in different areas of the field were selected for equidistant expansion polygon post-processing tests. Similarly, the proportion of frames in which leaves were completely recognized in the 500 frames before and after post-processing was used to represent the recognition completeness value of YOLOv8 and the recognition completeness value of equidistant polygon expansion post-processing. The maximum recognition completeness of YOLOv8 was 95.3%, and the average was 80.4%. After post-processing, the maximum recognition completeness was 97.1%, and the average was 86.4%. It can be seen that in the real field environment, the equidistant expansion polygon post-processing algorithm effectively improves the integrity and accuracy of leaf recognition.

**Figure 21:**
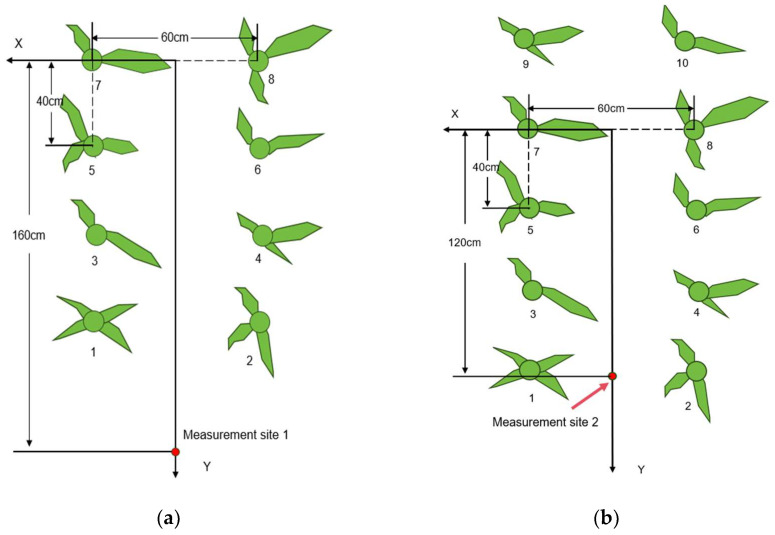
Field test schematic. (**a**) Schematic diagram of measurement point 1; (**b**) Schematic diagram of measurement point 2.

In the indoor test, it was clear that the measurement results within the range of 30 cm were limited by the measurement characteristics of the sensor, and there was a large error in the measurement results. Therefore, in the corn field test, only the leaves outside the 30 cm range of the test prototype were measured. Select corn planting rows with flat terrain and use laser displacement sensors to perform discrete measurements on leaves with good growth and measurement significance on both sides of the rows. The measurement results of the laser displacement sensor are also used as a reference for the optimal confidence point cloud measurement error. In total, 8 consecutive corn plants were selected between the rows for the test, and 10 measurement sites were selected for the test. The test schematic is shown in the Figure 21, where the red dot is measurement site 1, and the 10 measurement sites are evenly distributed on the *y*-axis with a spacing of 40 cm. For measurement site 1, plants 1 to 8 are the main measurement objects, and for measurement site 2, plants 3 to 10 are the main measurement objects. The measurement objects of measurement sites 3 to 10 are similar. During the test, UWB equipment is used to ensure the accuracy of the measurement site position.

**Figure 22:**
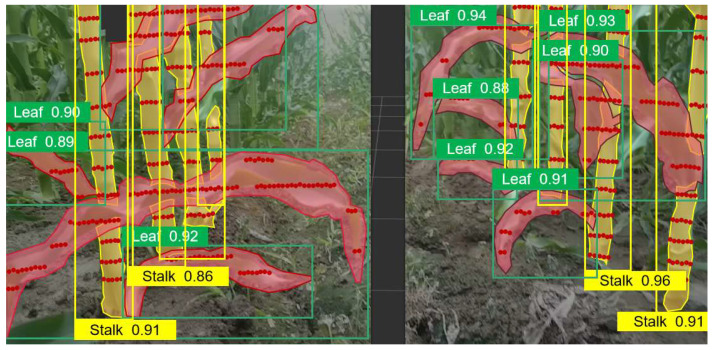
The interface of the host computer Rviz during the test.

The number of effective leaves in the 10 measurements was 308. The interface of the host computer Rviz during the test is shown in Figure 22. To enhance the recognition and clarity, the depth camera point cloud image is not shown in the figure. The red outline is the post-processing result of the equidistant expansion polygon, that is, the continuously identified leaves. The yellow outline is the clustering result of the stems under the FILL-DBSCAN algorithm. After screening the objects outside the measurement range and removing the abnormal points, the laser displacement sensor measurement results and the optimal confidence point cloud measurement results are compared with the distance measurement mean error and the convex hull feature point error. The optimal confidence point cloud distance measurement mean error is 2.9 cm, and the maximum error of the convex hull feature point is 6.3 cm. After that, only the laser radar or depth camera is used to measure the leaves. The distance measurement mean error is 8.8 cm, and the convex hull feature point error is 9.1 cm. As the measurement distance increases, the depth camera measurement error gradually increases. It can be seen that the optimal confidence point cloud can effectively improve the distance measurement accuracy of corn leaves. The main reason why the accuracy of the optimal confidence point cloud distance measurement in the corn field is lower than that in the laboratory is that the different flatness of the ground leads to a low coincidence between the laser displacement sensor and the test prototype measurement benchmark. At the same time, the field is more susceptible to natural wind and light, which will also increase the measurement error.

Clustering was achieved in all 26 plants with the support of the FILL-DBSCAN algorithm. Different from laboratory tests, the test prototype will measure corn plants at different positions when driving between corn rows. Even if the test prototype is in a certain position, the stalks cannot be clustered due to the occlusion of leaves on the stalks, but when the prototype drives to a position with less occlusion, the stalks can still be clustered. The measurement of the same corn stalk at different positions effectively reduces the impact of the occlusion effect of leaves on corn stalk identification.

## 4. Conclusions

This paper proposes a corn feature recognition and ranging algorithm based on the fusion of depth camera, multi-line LiDAR, and IMU. Firstly, the algorithm utilizes YOLOv8 to identify features such as corn stems and leaves during robot operation in cornfields. Considering significant morphological variations caused by leaf curling in natural growth conditions, an equidistant polygon expansion post-processing algorithm is proposed to connect and ensure the accuracy and effectiveness of leaf recognition results. Secondly, aided by IMU, the algorithm integrates depth camera and multi-line LiDAR, introducing the concept of optimal confidence point cloud to obtain accurate ranging point clouds in complex field scenarios with varying illumination conditions. This achieves precise ranging of corn features. Finally, addressing the discontinuous recognition of stems under leaf occlusion, the FILL-DBSCAN algorithm is used for secondary clustering of stems, thereby enabling accurate recognition and ranging of surrounding corn features during robot operation in cornfields. Experimental results show that the leaf recognition accuracy exceeds 95.61%, and the average stem recognition accuracy exceeds 95.8%. Moreover, it achieves submillimeter ranging errors beyond 30 cm, meeting the basic requirements for robot navigation in cornfields with low crop damage rates, thus ensuring corn yield and quality by laying the foundation for high-precision path planning and navigation of robots in agricultural fields.

## Figures and Tables

**Table 1:** Experimental conditions for leaf-equidistant polygon expansion post-processing.

Experimental Group	Gripper Rotation Angle (°)	Translation Distance (cm)
1	−90	−9
2	−75	−7.5
3	−60	−5
4	−45	−4.5
5	−30	−3
6	−15	−1.5
7	0	0
8	15	1.5
9	30	3
10	45	4.5
11	60	6
12	75	7.5
13	90	9

**Table 2:** Laser displacement sensor measurements at different measurement points.

Measurement Point	Number of Valid Distance Points n	Average Distance Ave (cm)	Convex Hull Feature Point Distances d (cm)			
			Left	Right	Top	Bottom
1	239	16.71	−1.92	19.26	12.77	28.71
2	251	46.87	38.59	48.14	47.68	49.28
3	246	79.25	81.85	85.01	79.93	85.01
4	240	124.3	123.91	123.53	128.16	123.53

**Table 3:** Measurement results of experimental prototype at different measurement points.

Measurement Point	Number of Valid Distance Points n		Average Distance Ave (cm)	Convex Hull Feature Point Distances d (cm)			
	LiDAR	Depth Camera		Left	Right	Top	Bottom
1	239	3512	13.61	−2.60	21.56	11.17	25.31
2	251	4088	46.37	38.39	48.66	47.91	48.83
3	246	3750	78.41	81.45	85.11	79.75	84.76
4	240	3599	125.12	123.61	123.43	128.16	123.79

## Data Availability

The authors confirm that the data supporting the findings of this study are available within the article.
